# An Evaluation of Point-of-Care HbA1c, HbA1c Home Kits, and Glucose Management Indicator: Potential Solutions for Telehealth Glycemic Assessments

**DOI:** 10.3390/diabetology3030037

**Published:** 2022-09-13

**Authors:** Dessi P. Zaharieva, Ananta Addala, Priya Prahalad, Brianna Leverenz, Nora Arrizon-Ruiz, Victoria Y. Ding, Manisha Desai, Amy B. Karger, David M. Maahs

**Affiliations:** 1 Division of Endocrinology, Department of Pediatrics, School of Medicine, Stanford University, Stanford, CA 94304, USA; 2 Stanford Diabetes Research Center, Stanford, CA 94304, USA; 3 Quantitative Sciences Unit, Division of Biomedical Informatics Research, Stanford University, Stanford, CA 94305, USA; 4 Department of Laboratory Medicine and Pathology, School of Medicine, University of Minnesota, Minneapolis, MN 55455, USA

**Keywords:** type 1 diabetes, pediatrics, HbA1c, telemedicine, continuous glucose monitoring

## Abstract

During the COVID-19 pandemic, fewer in-person clinic visits resulted in fewer point-of-care (POC) HbA1c measurements. In this sub-study, we assessed the performance of alternative glycemic measures that can be obtained remotely, such as HbA1c home kits and Glucose Management Indicator (GMI) values from Dexcom Clarity. Home kit HbA1c (*n* = 99), GMI, (*n* = 88), and POC HbA1c (*n* = 32) were collected from youth with T1D (age 9.7 ± 4.6 years). Bland–Altman analyses and Lin’s concordance correlation coefficient (*ρ*_c_) were used to characterize the agreement between paired HbA1c measures. Both the HbA1c home kit and GMI showed a slight positive bias (mean difference 0.18% and 0.34%, respectively) and strong concordance with POC HbA1c (*ρ*_c_ = 0.982 [0.965, 0.991] and 0.823 [0.686, 0.904], respectively). GMI showed a slight positive bias (mean difference 0.28%) and fair concordance (*ρ*_c_ = 0.750 [0.658, 0.820]) to the HbA1c home kit. In conclusion, the strong concordance of GMI and home kits to POC A1c measures suggest their utility in telehealth visits assessments. Although these are not candidates for replacement, these measures can facilitate telehealth visits, particularly in the context of other POC HbA1c measurements from an individual.

## 1. Introduction

For individuals with type 1 diabetes (T1D), hemoglobin A1c (HbA1c), self-monitoring of blood glucose (SMBG), and continuous glucose monitoring (CGM) are common metrics used to assess glycemic management. HbA1c has long been considered the gold standard in glucose monitoring [1]. Although HbA1c remains an important measure of glucose values over time and overall diabetes management, various conditions can impact the accuracy of HbA1c measures, including hemoglobinopathies, chronic kidney disease, or iron-deficiency anemia [1]. In routine clinical care, point-of-care (POC) HbA1c measurements using fingerstick samples with National Glycohemoglobin Standardization Program (NGSP) certification offer healthcare providers a method for real-time assessment of glycemic control [2].

With the increasing use of CGM technology [3], various CGM-derived estimations of HbA1c have evolved over the years. The term “estimated A1c” has also been replaced with terms such as Glucose Management Indicator (GMI) to reduce confusion if laboratory HbA1c and estimated A1c differ. CGM-derived glucose metrics offer promising, real-time, and effective data to better manage T1D [4] by focusing on the glucose time-in-range (i.e., time spent between 70–180 mg/dL).

CGM-derived metrics have become particularly relevant during the COVID-19 pandemic, where fewer in-person clinic visits resulted in fewer POC HbA1c measurements. Therefore, as reported by Beck and colleagues [5], there is an increased need for alternate methods to assess HbA1c, including academic laboratories’ fingerstick capillary blood collection kits suitable for home use (HbA1c home kit) and CGM-derived GMI [6]. This work aimed to assess the accuracy and concordance between POC HbA1c, HbA1c home kit, and GMI in pediatric patients with T1D within the 4T Study when the COVID-19 pandemic required creative solutions to monitor glucose control. We report these findings as they may expand options for evaluating diabetes care.

## 2. Materials & Methods

This sub-study was part of the larger 4T Study, which aims to initiate CGM (Dexcom G6; Dexcom, San Diego, CA, USA) shortly after T1D diagnosis, approved by the Stanford University Institutional Review Board (clinicaltrials.gov: NCT03968055 and NCT04336969) [7-16]. Inclusion criteria for the 4T Study included all youth within one month of the T1D diagnosis seen in the Stanford Lucile Packard Children’s Hospital ages six months to 21. A formal diagnosis of T1D included at least one positive autoantibody. The exclusion criteria for the 4T Study included a diabetes diagnosis other than T1D, diagnosis greater than one month before the initial study visit, individuals to obtain diabetes care at another clinic, individuals who do not consent to CGM use, and individuals greater than 21 years of age.

HbA1c measurements were collected from 71 youth with T1D (age 9.7 ± 4.6 years, 41% female, and 48% Non-Hispanic White, Table 1). Of the 71 unique participants, 23 (32.4%) contributed more than one HbA1c home kit measurement (University of Minnesota’s Advanced Research and Diagnostic Laboratory [ARDL]) for a total of 99 HbA1c values for which there were GMI (*n* = 88) and concurrent POC HbA1c (*n* = 32; DCA Vantage^®^ Analyzer, Siemens, Germany) to evaluate these three methods for measuring glycemic control. The ARDL HbA1c measurements were performed on the Tosoh G8 HPLC system in variant mode (Tosoh Bioscience, Inc., South San Francisco, CA, USA). Although there are a variety of methods for computing a HbA1c value from CGM data [17-19], we chose GMI in this study because all of the participants enrolled in the 4T Study were started on a Dexcom G6 CGM system, and we were able to determine GMI readily using Dexcom Clarity reports.

The HbA1c home kit and POC HbA1c (*n* = 32) measurements were collected on the same day in the clinic with the guidance of the study staff. The HbA1c home kit and GMI (*n* = 88) and POC A1c and GMI (*n* = 27) were also collected on the same day. The HbA1c home kit included an alcohol wipe, gauze, a single-use lancing device, a capillary tube, a Bio-Rad hemoglobin capillary collection vial with ethylenediaminetetraacetic acid (EDTA) and potassium cyanide; a cardboard stand to hold the collection vial, written instructions, a blood collection form, a biohazard bag, a gel pack for freezing, an absorbent pad, and a cardboard shipping box with a pre-paid return label. The HbA1c home kits were collected, packaged according to instructions, and mailed via United States Postal Service (USPS) as first-class mail to the ARDL at the University of Minnesota for analysis.

The POC vs. GMI only had 27 paired samples (versus 32 for POC vs. the home kit) due to missing GMI data in five participants. Similarly, for the home kit vs. GMI, there were 88 paired samples (of the 99 total home kits) due to the 11 participants missing GMI data. Overall, there were fewer matched comparisons available for the POC HbA1c because fewer families attended in-clinic visits during the COVID-19 pandemic. GMI values were captured on the same day the home kit and POC HbA1c were collected, and we used a 90-day retrospective GMI measurement period via Dexcom Clarity reports.

## 3. Statistical Analysis

Matched pairs were used for Bland–Altman analyses and Lin’s concordance correlation coefficient (*ρ*_c_) to evaluate the agreement among glycemic control measures. Paired measures include the following: POC vs. home kit = 32 paired; POC vs. GMI = 27 paired; and home kit vs. GMI = 88 paired samples. Bias was defined as the difference between the alternative glycemic control measure and the reference POC A1c as presented for each measure. When comparing home kits’ A1c and GMI, the home kit was used as the reference measure since the standard of care POC HbA1c was not available for these matched pairs. All matched HbA1c measurements were collected on the same day, and 90-day GMI data were captured using the CGM mean glucose value from Dexcom Clarity. To confirm that the observed differences fell within the equivalence bounds (i.e., between −0.5 and 0.5%), a TOST analysis for equivalency was conducted using paired *t*-tests.

## 4. Results

**Figure 1.**
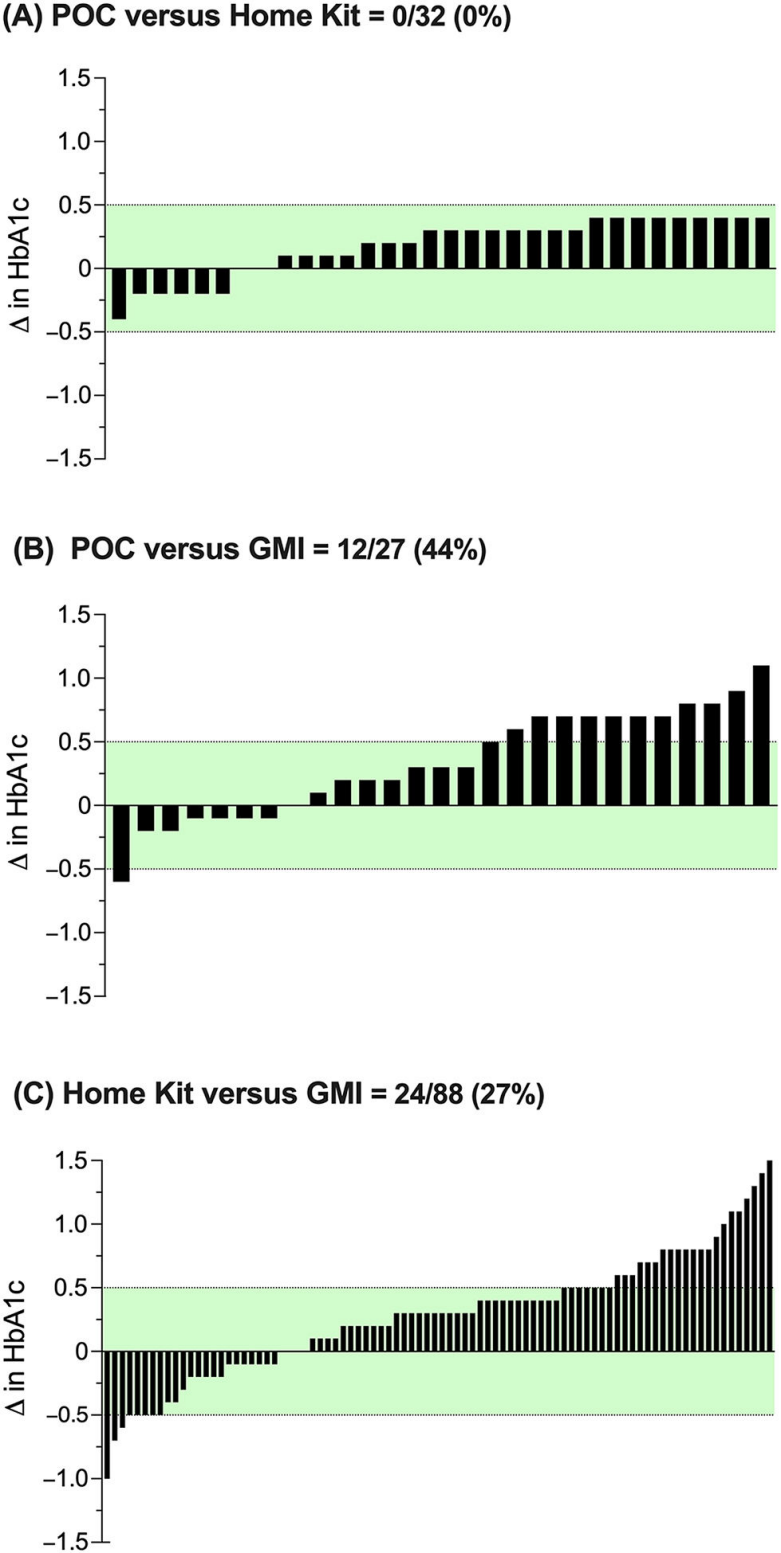
Bland–Altman comparison of the HbA1c home kit, GMI, and POC HbA1c measurements. The solid line (blue) represents the mean difference between comparator and reference, and the dashed lines (red) represent the 95% limits of agreement. In (**A**), the HbA1c home kit is the comparator, and POC HbA1c is the reference. In (**B**), GMI is the comparator, and POC HbA1c is the reference. In (**C**), GMI is the comparator, and HbA1c home kit is the reference. GMI = Glucose Management Indicator; POC = point-of-care HbA1c.

The median CGM wear time to evaluate GMI was 98.9% (IQR 86.7, 100%). In relation to POC HbA1c (Figure 1), both HbA1c home kit (panel (A)) and GMI (panel (B)) showed a slight positive bias (mean difference 0.18% and 0.34%, respectively). The HbA1c home kit and GMI showed strong concordance with POC HbA1c (*ρ*_c_ = 0.982 [0.965, 0.991] and 0.823 [0.686, 0.904], respectively). The GMI (panel (C)) also showed a slight positive bias (mean difference 0.28%) and fair concordance (*ρ*_c_ = 0.750 [0.658, 0.820]) with the HbA1c home kit.

**Figure 2.**
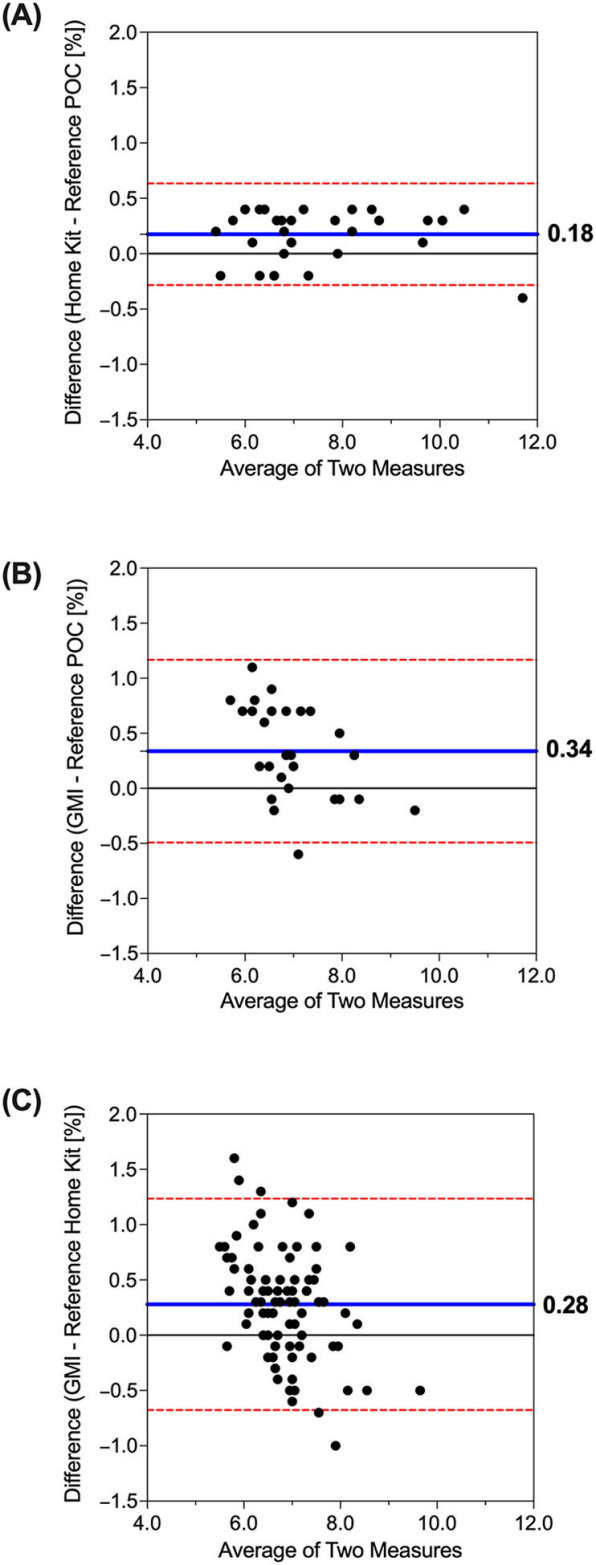
Paired HbA1c values are as follows: (**A**) POC versus Home Kit A1c, (**B**) POC versus GMI, and (**C**) home kit versus GMI. The green shaded area denotes HbA1c values that did not deviate by a clinically meaningful amount (>0.5%).

In addition, the percentage of the paired HbA1c values that deviated by a clinically meaningful amount (>0.5%) was 0% (POC versus home kit, 0/32), 44% (POC versus GMI, 12/27), and 27% (home kit versus GMI, 24/88); Figure 2. For each pairwise comparison, we tested the composite null hypotheses H0_1_: Δ ≤ −0.5 and H0_2_: Δ ≥ 0.5. Upon rejection at the 0.05 alpha level, we concluded that the observed differences fall within the predefined equivalence bounds of −0.5 and 0.5%.

The Bland–Altman analyses revealed that 30 and 90 days of GMI data showed slightly less bias than using 14-day GMI data (mean difference of 0.34% for both 30 and 90 days of the GMI data versus 0.38% for 14-day GMI data, respectively).

## 5. Discussion

In this sub-study, we assessed the accuracy and concordance between POC HbA1c, HbA1c home kit, and Glucose Management Indicator (GMI) values in pediatric patients with type 1 diabetes. The COVID-19 pandemic has led to changes and rapid adoption of diabetes care delivery in a telehealth model. Even with more openings, patients and families often choose telehealth for convenience [9]. Therefore, implementation and accessibility to HbA1c home kits will allow for regular glycemia and patient-centered care monitoring. This sub-study demonstrates that the HbA1c home kit and GMI show a strong concordance with the standard of care POC HbA1c, and these data support the use of the ARDL home HbA1c kits within the 4T study [7-16], as well as verified home HbA1c kits as an option for clinical care for telehealth diabetes care. Beck et al. [5] recently reported similar findings with two capillary blood collection kits (one of which was the ARDL HbA1c home kit described here) and venous HbA1c. They show compelling evidence that HbA1c measurements from these two capillary blood collection kits can be used interchangeably with venous HbA1c. In our analysis, we extend these findings and demonstrate the added utility of GMI in youth wearing CGM technology.

There are limitations in this sub-study worth noting. We used the Dexcom G6 CGM system in the present study, so these conclusions may not be generalizable across different types of CGM systems. In addition, our POC reference device used in this analysis was the DCA Vantage analyzer based on latex immunoagglutination inhibition, a commonly used POC A1c measurement system. Different POC HbA1c instruments may work by different methodologies (e.g., boronate affinity chromatography); therefore, we also note the lack of generalizability of these conclusions across other HbA1c POC systems that were not tested. Similarly, in this sub-study, we obtained a relatively small number of matched pairs (specifically for POC measurements due to the COVID-19 pandemic). This may warrant a more robust analysis to confirm the current findings.

The use of telehealth-specific options for A1c measures (i.e., GMI and home kit) has its own unique set of pros and cons. In our analysis, the A1c home kit outperformed GMI in concordance. However, the A1c home kit also requires additional collection kit instructions for patients, the possibility of user error in sample collection, and challenges around timely shipment and analysis of samples. GMI calculations do not correct glycation rates, and studies have shown that similar methods may yield more accurate A1c estimations [17-19]. Therefore, future studies might consider different methods for calculating HbA1c from CGM tracings that may correct the rate of glycation and, in turn, the lifetime of red blood cells. The convenience of GMI is that it does not require additional testing or associated costs like the A1c home kit and is obtainable from the CGM device already in use.

## 6. Conclusions

In conclusion, these data demonstrate that the HbA1c home kit and GMI show strong concordance with POC HbA1c. The use of GMI data in this analysis is not intended to replace future POC or venous blood samples for HbA1c. However, using GMI may be particularly helpful for families and individuals that may not have access to POC HbA1c or the HbA1c home kit and to facilitate telehealth visits. CGM data also provide additional information on hypoglycemia and glucose variability [20]. Overall, the HbA1c home kit and GMI may be potential solutions to glycemic assessment during the COVID-19 pandemic and for future telehealth visits.

## Figures and Tables

**Table 1.** Participant Demographics. Note: N = 71 unique participants (25 contributed more than one measurement).

Demographics	Mean ± SD
	Median [IQR]
**Age, years**	9.7 ± 4.6
	10 [6–14]
**Sex**	
Male	29 (41%)
Female	42 (59%)
**Race/Ethnicity**	
Non-Hispanic White	34 (48%)
Non-Hispanic Black	0 (0%)
Hispanic	7 (10%)
Asian/Pacific Islander	10 (14%)
Other	2 (3%)
Unknown	18 (25%)
**Body Mass Index (BMI)**	18.6 ± 6.1
	17 [15–21]
**Insurance Type**	
Private	55 (77%)
Public	16 (23%)
**HbA1c at Diagnosis (%)**	12.5 ± 2.2
**HbA1c Home Kit (%)**	6.9 ± 1.2
**Months Since Diagnosis**	8.6 ± 5.1
	8 [4–12]

## Data Availability

Data is contained within the article.
